# Stereotactic radiosurgery with Cyberknife^®^: first case in the United Arab Emirates (a case report)

**DOI:** 10.11604/pamj.2022.42.267.33358

**Published:** 2022-08-10

**Authors:** Nandan Maruti Shanbhag, Christos Antypas, Abdul Karim Msaddi, Teekendra Singh, Sinead Catherine Murphy, Benjie Baguitan

**Affiliations:** 1 Neuro Spinal Hospital, Dubai, United Arab Emirates

**Keywords:** Stereotactic radiosurgery, Cyberknife^®^, precision radiotherapy, case report

## Abstract

A 64-year-old gentleman was referred to the department of oncology with severe pain in the right ear radiating to the right side of the face. Imaging revealed a large extra-axial expansile lesion, surrounding and encasing the right cavernous sinus extending to the right middle cranial fossa. The patient consulted several neurosurgeons and was recommended stereotactic radiosurgery with Cyberknife^®^ as the best non-invasive modality. The proximity to the critical structures, such as the brainstem, made it challenging for any surgical approach. The patient completed stereotactic radiosurgery with Cyberknife^®^ and is doing well one month after treatment.

## Introduction

With 4807 new cases registered in 2020, cancer is among the leading causes of mortality in the United Arab Emirates [1]. Tumours of the nervous system are a part of new cases being diagnosed every year, with many of them difficult to manage either due to their size or proximity to the critical structures. Cavernous sinus mass has a wide variety of differential diagnoses with meningioma followed by schwannomas of the cranial nerves.

Surgery continues to be the primary modality in many cases but is associated with higher rates of neurovascular damage and sometimes massive bleeding [2]. In such instances, stereotactic radiosurgery has emerged as the primary modality of treatment that can be delivered with precision accuracy and safely utilising machines such as Cyberknife^®^ [3]. Cyberknife^®^ is a non-invasive treatment for benign and malignant conditions where radiation therapy is indicated. The case was reviewed during the multidisciplinary meeting (tumour board), the benefits and risks of the various treatments were discussed and agreed to the treatment plan - stereotactic radiosurgery with Cyberknife^®^.

The indication for Cyberknife^®^ was two-fold, firstly the proximity of the tumour to the critical structures, secondly, the surgical risks involved. The patient's written informed consent was obtained after discussing the treatment plan, including the treatment safety, adverse effects, and other alternative treatment options. We also considered the patient preference for a non-invasive modality as the patient was sceptical about the surgical interventions due to the risks involved.

## Patient and observation

The patient was well until six months ago when he developed severe pain in the right ear radiating to the right side of the face. He describes the pain as being “sharp shooting pain” and radiating to the lower part of the face. The pain was a 9/10 on a pain scale [4]. This he noticed was exaggerated after he extracted his last upper molar on the right side. He immediately consulted a neurologist who referred him to a neurosurgeon, and imaging confirmed an extra-axial mass in the proximity of the right cavernous sinus. The patient then was explained that any surgical intervention, including a biopsy, was risky given the proximity of the lesion to the critical structures. The patient has been hypertensive on medications for more than 15 years, related to myocardial infarction with a history of multiple stents. Hyperlipidemia on medications and most recently has recovered from COVID-19. He was incidentally diagnosed with an aortic aneurysm during the workup for SARS-CoV-2 infection. He also has a history of cataracts. He has been treated for prostatitis. He had lower back surgery for a lump excision, probably a lipoma.

**Clinical findings:** there were no significant clinical findings on examination and no neurological deficits.

**Figure 1:**
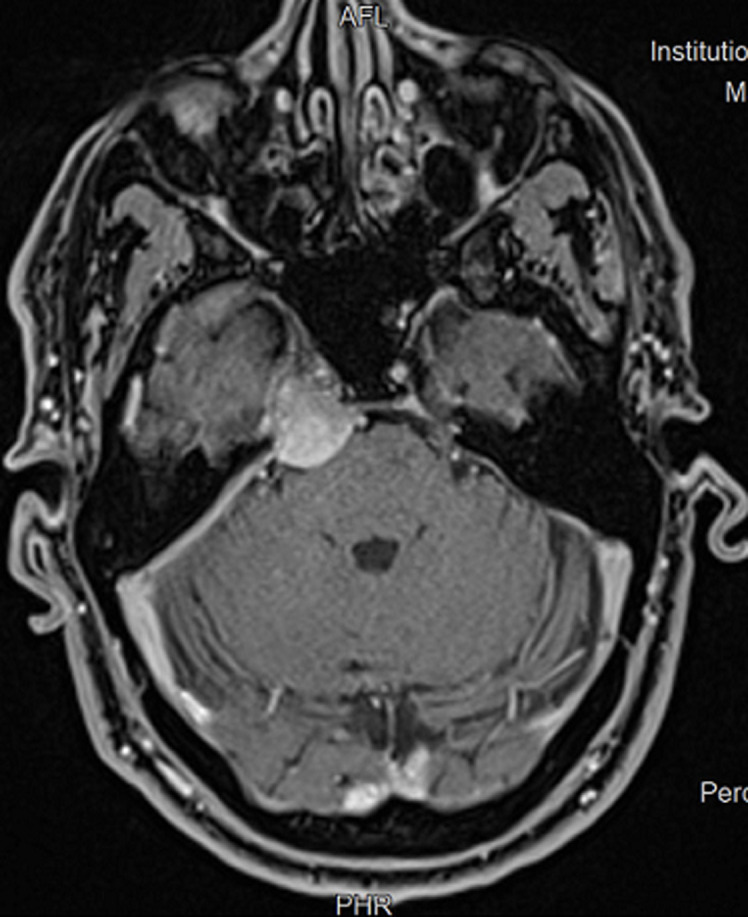
T1 MRI with contrast (coronal) showing the mass near the right cavernous sinus

**Diagnostic assessment:** a well-defined extra-axial soft tissue lesion measuring 26 x 20 x 24 mm is seen around the right cavernous sinus encasing the vessels without affecting its lumen. It is seen paralleling the grey matter intensity signal, relatively effacing the right side of the pons with diffuse homogeneous post gadolinium enhancement and dural tail enhancement extending overlying the right side of the clivus bone. There is no extension within the right internal auditory canal (yet just reaching its outer edge) (Figure 1).

**Diagnosis:** probable differentials include meningioma (most probable), schwannoma of the cranial nerves and very unlikely a carcinoma, inflammation, or a hemorrhagic process.

**Figure 2:**
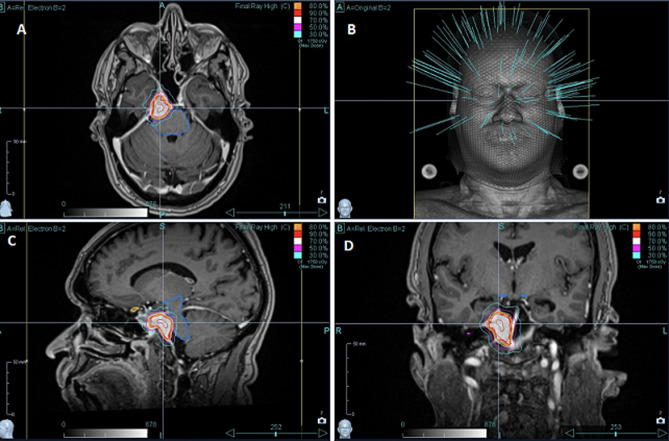
(A,B,C,D) the Cyberknife^®^ Accuray^®^ precision plan, isodose and dose volume histogram

**Therapeutic interventions:** the treatment included simulation, contouring, plan generation, plan quality assurance plan approval and treatment delivery. During simulation, the patient was immobilized with a customized, non-invasive 2.4 mm thermoplastic mask formed over the face and affixed to an acrylic baseplate frame attached directly to the cyberknife^®^ couch. For patient comfort, a custom-formed pillow (AccuForm™, Civco Medical Systems, Orange City, USA) was used and shaped around the base of the head to hold its shape indefinitely. The non-contrast computed tomography (CT) scan was fused with contrast-enhanced CT and the T1 weighted magnetic resonance imaging (MRI), both with 1 mm slice thickness, to delineate the target and the organs at risk. Plan evaluation was done with a close observation of the Isodose distribution and the dose volume histogram and approved after the set parameters were met (Figure 2).

For treatment delivery with image guidance, 6D skull tracking mode was used. At the same time, the patient was positioned on the treatment Robo-couch in the same position as during simulation, using in-room lasers. For initial patient alignment, orthogonal kV X-ray pairs were acquired using an in-room imaging system and compared with the planning digitally reconstructed radiographs (DRR) using the 2D-3D image registration method [5]. Initial set-up errors were detected and corrected by Robo-couch movement, thus bringing the patient to the treatment position. After the treatment started, real-time deviations were continuously recorded and adjusted by the robotic arm. The robot can automatically correct up to ±10 mm in all translational axes and up to ±1.5 degrees in all rotational axes without moving the patient [6,7]. Imaging interval varied between 20 seconds up to 45 seconds depending on the patient´s movement, minimizing intrafraction inaccuracy. A dose of 14Gy (80%) was delivered in a single fraction.

**Follow-up and outcome of interventions:** the patient was followed up in 3 weeks and is doing well with no adverse reactions, and the pain has significantly subsided.

**Patient perspective:** “I have been diagnosed as a patient with a benign brain tumour, and all the neurosurgeons that i have met advised of stereotactic radiation surgery as the most suitable treatment in such case. I got treatment by the cyber-knife robotic radiation surgery which is a very advanced technique in such a case. During the procedure, I felt no pain nothing just lying down with no movement until they told me that it is finished, for me, it is an amazing treatment as I didn't expect it to be such easy, and after the treatment, I walked normally and even my family when I met them and saw me walking normally and talking to them, they didn't believe it. Now, after the treatment, I practice my life normally, and no side effects are observed. My perspective about the treatment is positive, and the most important for me is that it is safe and with no risk”.

**Informed consent:** the patient signed a written informed consent.

## Discussion

The clinical management of a patient with central nervous tumours is challenging. This is primarily due to the proximity to the critical structures. In cases such as those close to or involving the cavernous sinus, there is fear of causing massive bleeding in addition to the damage caused to the nerve structures leading to permanent morbidity. With excision being risky, stereotactic radiation techniques - stereotactic radiosurgery (SRS) and stereotactic radiotherapy (SRT) - have emerged as safe modalities, either adjuvant or in some instances as the primary modality with 5-yr progression-free survival rates close to 90% [8]. More importantly, post-treatment neurological deficits have been low, and preservation rates close to 100% [9].

Stereotactic radiosurgery (SRS) delivers a high radiation dose in a single sitting. At the same time, stereotactic radiotherapy (SRT) or hypofractionated stereotactic radiotherapy refers to the delivery of radiation (usually more than 5Gy) in divided doses [10]. SRS is either delivered as adjuvant therapy or even as a primary modality in early grade or benign World Health Organization (WHO) tumours, including meningiomas, as the local control is similar. There is no standard grading system to guide a clinician for treatment selection as either SRS or SRT. This largely depends on the size of the tumour, location, and proximity to critical structures. In general, a cavernous sinus meningioma, well contained within the region and size not exceeding 3 cm, SRS is preferred and safely delivered. For a larger tumour enclosing the eloquent structures, SRT is preferable [11]. The dose delivered with SRS ranges from 11Gy - 19Gy for WHO grade 1 meningiomas, and some studies have supported lower marginal doses of 12Gy for benign cavernous sinus tumours [12,13].

Cavernous sinus meningiomas larger in size, hypofractionated stereotactic radiotherapy is preferred as it provides equivalent tumour control and lesser side effects than SRS [14-16]. The ideal follow-up and response time for the cavernous sinus meningiomas has been a matter of debate. Some of the tumours have been known to remain stable for almost two years before regression. As a general clinical practice, the patient is followed up three weeks after the treatment and then every three months for the first year and every six months for the following two years and then once a year for regular follow-up. Imaging studies are done three months after the treatment and at the end of 1 year to assess response to treatment [17,18]. Radiological and clinical examinations, including neurological and ophthalmological assessments, could be extended to a longer time interval depending on the initial follow-up results and patient symptoms.

## Conclusion

Central nervous tumours provide a comprehensive set of challenges for treatment due to the presence of critical structures in proximity. Often, surgery risks damaging these structures and may lead to permanent morbidity. Novel treatment techniques like stereotactic radiosurgery with the Cyberknife^®^ robotic system are emerging as primary treatment modalities and must be explored further. This is the first case treated with stereotactic radiosurgery with Cyberknife^®^ in the United Arab Emirates and demonstrates that such treatments can be delivered with safety and precision.
